# 影像组学在非小细胞肺癌免疫治疗中的研究进展

**DOI:** 10.3779/j.issn.1009-3419.2024.102.29

**Published:** 2024-08-20

**Authors:** Yue HOU, Tianming ZHANG, Hong WANG

**Affiliations:** 730000 兰州，兰州大学第二医院呼吸内科; Department of Respiratory Medicine, Lanzhou University Second Hospital, Lanzhou 730000, China

**Keywords:** 影像组学, 肺肿瘤, 免疫治疗, Radiomics, Lung neoplasms, Immunotherapy

## Abstract

肺癌是肿瘤相关死亡的主要原因，非小细胞肺癌是其中最常见的亚型。目前，以程序性细胞死亡受体1或其配体的免疫检查点抑制剂为代表的免疫治疗已广泛应用于非小细胞肺癌患者的临床诊疗实践，但仅少数患者能从中受益，免疫治疗缺乏可靠的预测标志物。影像组学是一种借助计算机软件使用数据表征算法从生物医学图像中提取大量定量信息的工具，大量研究已经证实预测非小细胞肺癌免疫疗效的影像组学模型作为新型免疫疗效预测标志物，有望指导肺癌患者的个体化诊疗决策，具有光明的应用前景。本文对影像组学预测非小细胞肺癌免疫治疗反应、识别假性进展和超进展、免疫检查点抑制剂相关性肺炎、恶病质风险和联合其他组学中的研究进展作一综述。

## Abstract (en)

Lung cancer is the main cause of cancer-related deaths, with non-small cell lung cancer (NSCLC) being the predominant subtype. At present, immunotherapy represented by immune checkpoint inhibitors (ICIs) of programmed cell death receptor 1 or its ligand has been widely used in the clinical diagnosis and treatment of patients with NSCLC. However, only a few patients can benefit from it, and reliable predictive markers for immunotherapy are lacking. Radiomics is a tool that uses computer software and algorithms to extract a large amount of quantitative information from biomedical images. A large number of studies have confirmed that the radiomic model that predicts the immune efficacy of NSCLC can be used as a new type of immune efficacy predictive marker, which is expected to guide the individualized diagnosis and treatment decisions for patients with lung cancer and has a bright application prospect. This article reviews the research progress of radiomics in predicting the immune therapy response of NSCLC, identifying pseudo-progression and hyperprogression, ICIs-related pneumonia, cachexia risk, and combining with other genomics.

肺癌的发病率和死亡率现已跃居世界恶性肿瘤首位，其中，非小细胞肺癌（non-small cell lung cancer, NSCLC）约占肺癌病理亚型的85%^[1,2]^。近年来，多个针对程序性细胞死亡受体1（programmed cell death 1, PD-1）或其配体（programmed cell death ligand 1, PD-L1）的免疫检查点抑制剂（immune checkpoint inhibitors, ICIs）获批写入肺癌诊疗指南，临床医生制定个体化治疗方案有了可靠依据^[3]^。免疫治疗效果与肿瘤异质性、肿瘤微环境、PD-L1的动态变化等多种因素有关，目前临床主要应用免疫组织化学检测PD-L1的表达情况。通过组织活检检测PD-L1因其有创性及依从性差导致应用受限，同时，PD-L1表达的动态变化无法监测，且肿瘤组织PD-L1表达具有异质性。Munari等^[4]^评价了PD-L1检测的可靠性并指出，至少4次以上组织活检才能减少肿瘤PD-L1表达的错误分类，基于1次PD-L1检测结果制定抗癌方案时应谨慎。最近发表的一项研究^[5]^汇总分析了KEYNOTE-189及其日本队列、KEYNOTE-407及其中国队列中PD-L1阴性（PD-L1<1%）晚期NSCLC患者5年随访结果，分析指出帕博利珠单抗联合化疗对于PD-L1阴性的未经治疗的转移性NSCLC患者的生存结果优于单纯化疗。由此可见，免疫治疗并非PD-L1阳性（PD-L1≥1%）肺癌患者的专属治疗，如何优化患者免疫治疗生存获益预测的难题亟待解决。人工智能依托下影像组学的飞速发展为攻克这一临床难题奠基了坚实基础。本文对影像组学在NSCLC患者免疫治疗的疗效、预后、ICIs相关性肺炎（ICIs ralated pneumonia, CIP）、恶病质风险和联合其他组学中的研究进展作一综述，旨在报告和讨论影像组学特征作为生物标志物在肺癌领域的主要发现，有助于临床决策并改善患者预后。

## 1 影像组学的原理及简要过程

影像组学（radiomics）是一种借助计算机软件使用数据表征算法从生物医学图像中提取大量定量信息的工具，其假说^[6,7]^认为肿瘤在遗传和分子水平上发生的与组织基因型和表型相关的特征在宏观影像上有所体现，影像组学的原理^[6]^则是建立在假说基础上，运用图像定量分析技术挖掘人眼无法识别的影像组学特征，随后使用统计和机器学习（machine learning）对这些数据进行分析并与有意义的临床指标进行相关性分析并建立预测模型，从而探索能够预测患者预后的影像学标志物。其基本过程^[7,8]^包括医学影像图片的获取、感兴趣区域病灶分割、影像组学特征提取与筛选、构建影像组学模型、模型的验证。早期肺癌最常见的病理类型是肺腺癌，常以肺磨玻璃结节（ground glass nodules, GGNs）为主要表现形式，Shi等^[9]^指出基于影像组学的人工智能定量参数在一定程度上能够区分GGNs的病理类型，为临床开展精准化治疗提供参考依据。NSCLC患者肿大淋巴结的良恶性诊断关系后续治疗手段的选择及预后判断，有研究^[10]^认为基于影像组学构建的NSCLC患者肿大淋巴结良恶性诊断模型表现出巨大的应用价值。迄今为止，影像组学在肺癌早期诊断、基因表型识别及治疗评价等各阶段^[11]^广泛应用。

## 2 影像组学在NSCLC免疫治疗中的应用

### 2.1 预测基因表型及免疫检查点

基因表型识别是肺癌治疗的必经之路，现有检测手段如免疫组织化学、聚合酶链式反应及下一代宏基因组测序都存在有创且相对昂贵的问题。

Mu等^[12]^纳入来自多中心的697例NSCLC患者开发深度学习评分（deep learning score, DLS），研究指出DLS可显著区分PD-L1阳性和阴性患者，DLS在训练集、验证集和外部验证集的受试者工作特征（receiver operating characteristic, ROC）曲线下面积（area under the curve, AUC）均≥0.82；研究还指出在预测肿瘤进展时间和总生存率方面，DLS和基于免疫组织化学检测的PD-L1评分无显著差异。此外，诸多影像组学模型^[13⇓⇓⇓⇓-18]^预测肺癌常见基因突变及PD-L1分层表达时优于临床因素预测模型，强有力地证实了影像组学能够辅助临床医生制定不同的治疗方案。但无创性影像组学模型预测PD-L1表达能否替代免疫组织化学检测等病理金标准仍需更多前瞻性试验。

### 2.2 预测NSCLC患者免疫治疗反应

NSCLC的疗效与影像组学特征显著相关，Prelaj等^[19]^系统评价了90项利用人工智能确定肿瘤免疫治疗获益的预测标志物的研究并指出，大多数研究均显示使用人工智能开发的免疫治疗获益的预测标志物具有潜在应用前景，但仍缺乏新预测标志物能够即刻改变临床实践的有力证据。相比耗时费力的传统阅片，经济高效的人工智能具有更加广阔的应用前景，应用影像组学开发无创易获取的生物预测标志物是肺癌精准治疗的有效途径。

已有研究^[20⇓⇓-23]^证实应用影像组学特征或深度学习（deep learning）探索免疫治疗效果的预测标志物是可行的，但这些研究局限于仅针对表皮生长因子受体（epideral growth factor receptor, EGFR）突变、CD8^+ ^T细胞浸润、肿瘤突变负荷（tumor mutation burden, TMB）、淋巴细胞浸润、PD-L1表达的预测从而间接预测免疫治疗反应，对影像组学特征是否可以独立应用于预后预测的问题没有探讨。Saad等^[24]^探索预测免疫抑制剂起效的影像特征并评估其临床应用价值，进行了回顾性建模分析。研究从两家癌症中心3428例晚期NSCLC患者中筛选纳入976例接受ICIs单药或联合化疗的EGFR/间变性淋巴瘤激酶（anaplastic lymphoma kinase, ALK）野生型患者，且计算机断层扫描（computed tomography, CT）图像均采集于免疫治疗开始前3个月以内，应用深度学习从CT图像提取影像组学特征并联合现有的临床病理标志物构建复合模型。结果显示，单因素分析复合模型预测性能显著提高，总生存期（overall survival, OS）的C指数从0.7（临床模型）增加到0.75（复合模型），但单独的影像组学无法达到深度学习的疗效预测性能，研究表明应用深度学习对CT图像进行自动分析可以独立预测预后。Saad等^[24]^的研究是第一个反映大规模真实世界人群的影像组学研究，纳入患者的CT图像采集自不同的机器和成像技术，在多中心研究中模拟了不同数据，提高了模型的通用性，向NSCLC患者精准免疫治疗目标迈进一步。多项研究^[25⇓⇓⇓⇓-30]^已经证实了影像组学在晚期NSCLC免疫疗效预测中的潜在应用价值。

#### 2.2.1 预测早期免疫治疗反应

术前新辅助免疫化疗为NSCLC的治疗策略带来革命性变化，Qu等^[31]^纳入248例来自上海瑞金医院、宁波华美医院和遵义医科大学附属医院多家机构接受新辅助免疫化疗及手术的NSCLC患者，回顾性收集患者术前新辅助免疫化疗前2周内的CT图像并运用深度学习开发预测模型。整体人群中29.4%的病例获得病理完全缓解（pathologic complete response, pCR），在内部验证集和外部验证集中，模型对pCR预测的AUC分别为0.775（95%CI: 0.649-0.901）和0.743（95%CI: 0.618-0.869），显著优于临床模型的0.579（95%CI: 0.468-0.689）和0.569（95%CI: 0.454-0.683）。Qu等^[31]^开发的深度学习模型对NSCLC患者新辅助免疫化疗的pCR预测能力良好，研究还指出更高的模型得分与细胞代谢通路的上调和更多的微环境中抗肿瘤免疫浸润有关。

Delta-radiomics是影像组学的一个分支，主要研究影像组学特征在时间上的变化。Barabino等^[32]^对33例接受ICIs治疗NSCLC患者的基线及首次疗效评估的增强CT进行分析，确定了27个可以预测ICIs治疗反应的delta影像组学特征，且研究显示其中9个特征的变化与假性进展（pseudo-progression, PsPD）显著相关。Gong等^[33]^利用短期随访CT开发影像组学模型预测晚期NSCLC免疫治疗反应的研究表明，与免疫治疗前CT影像组学模型（pre-radiomics model）相比，基于治疗前后CT影像组学模型（delta-radiomics model）表现出更好的预后预测能力。研究纳入上海肺科医院和复旦大学附属肿瘤医院两家机构224例NSCLC患者并分为试验集、内部验证集和外部验证集，纳入患者均为III-IV期且大部分患者接受常规化疗后以单纯免疫治疗作为二线及后线治疗，并根据实体瘤疗效评价标准（Response Evaluation Criteria in Solid Tumors, RECIST）将完全缓解和部分缓解定义为免疫应答组，将疾病稳定和疾病进展定义为免疫无应答组。其中，试验集包含上海肺科医院93例患者（应答者34例，无应答者59例），内部验证集包含上海肺科医院63例患者（应答者24例，无应答者39例），外部验证集包含复旦大学附属肿瘤医院68例患者（应答者15例，无应答者53例）。免疫治疗前CT图像均在免疫治疗开始前1周内，第一次随访CT均在免疫治疗后6-8周。使用ITK-SNAP软件勾画肿瘤最大径作为感兴趣区域，提取出共1118个CT影像组学特征，应用支持向量机开发机器学习模型以预测免疫应答反应，最后应用Kaplan-Meier生存分析评估模型预测价值。结果显示，在验证集中，Delta-radiomics模式将ROC的AUC分别从0.64和0.52提高至0.82和0.87，差异具有统计学意义（P<0.05）。亚组分析还指出Delta-radiomics模式在腺癌中的预测效能优于鳞癌。Liu等^[34]^纳入197例NSCLC患者，提取ICIs治疗前及治疗1个周期后的影像组学特征并联合远处转移相关临床风险因素建立预测模型，同样证明了影像组学在早期免疫反应的评估中具有潜在优势。

上述研究均证明了影像组学在预测NSCLC患者早期免疫治疗反应中的应用价值。但术后接受免疫治疗的NSCLC患者因肿瘤病灶的缺失使得影像组学应用受限，此类患者仅适用基线影像组学预测OS，其基线影像组学的预测价值是否优于PD-L1表达检测仍需更多研究证实。此外，Qu等^[31]^的研究缺乏大规模前瞻性研究的支持，未来基于Delta-Radiomics预测接受术前新辅助免疫化疗患者的早期免疫治疗反应及远期预后方面尚待研究。

#### 2.2.2 预测免疫治疗远期疗效

临床上对患者免疫治疗远期疗效的评估主要来自以下几个指标，持久临床获益（durable clinical benefit, DCB）为根据RECIST评估的持续时间>6个月的部分缓解或疾病稳定；OS为免疫治疗开始至末次随访或死亡日期；pCR为肿瘤组织和淋巴结中均未观察到存活的肿瘤细胞；主要病理缓解（major pathological response, MPR）为原发病灶中活肿瘤细胞残留≤10%。Wu等^[35]^回顾性收集319例IB-IV期NSCLC患者接受免疫治疗前CT图像，组成训练集（n=214）、内部独立验证集（n=54）和外部验证集（n=51），以DCB和OS分别为远期疗效的主要预测指标和次要预测指标；同时在另一组98例术前接受ICIs治疗的可切除NSCLC患者中评估影像组学特征对病理反应的预测价值，以pCR和术前客观缓解率（objective response rate, ORR）为病理验证集（n=98）的预测指标。结果显示，模型在训练集和验证集均表现出良好的区分DCB和非DCB患者的预测能力，AUC分别为0.82（95%CI: 0.75-0.88）和0.75（95%CI: 0.64-0.87）；且模型对OS和无进展生存期（progression-free survival, PFS）的分量能力表现良好；亚组分析显示模型的预测价值显著优于PD-L1表达或单纯临床模型，同时还指出PD-L1分层表达与患者总生存率无关。Wu等^[35]^研究建立的预测模型能够作为区分ICIs治疗后DCB及非DCB患者的无创性预测标志物，用于支持晚期和可切除NSCLC是否使用ICIs治疗临床决策。

Farina等^[36]^纳入来自两家机构的246例受试者并收集免疫治疗第1和3周期CT图像及血清学数据，预测免疫治疗的NSCLC患者DCB，研究开发出集成深度影像组学特征和临床数据的模型并验证了模型对NSCLC患者ICIs治疗持久获益预测效能，结果显示模型在试验集和验证集中对预测ICIs治疗后6和9个月的AUC分别为0.824和0.753，模型预测效能较传统影像组学模型更可靠；研究还发现43.9%的患者接受ICIs治疗6个月后对免疫治疗仍反应，而9个月后反应率仅剩33.2%。基于先前的研究^[12,37,38]^可以认为深度学习技术能够提取出更高层级的与免疫治疗反应关系密切的空间特征，Farina等^[36]^同样证实了这一观点。

Wu等^[39]^则基于平扫CT（non-contrast enhanced CT, NCE-CT）和增强CT（contrast enhanced CT, CE-CT）分别建立联合远处转移临床因素的影像组学模型，研究纳入7家机构131例接受ICIs治疗的NSCLC患者。结果显示，采用原发病灶建立的NCE影像组学和CE影像组学在预测免疫治疗反应方面表现不佳，而基于最大病灶建立的NCE影像组学和CE影像组学均显示出令人满意的结果，验证集AUC分别为0.78（95%CI: 0.64-0.92）和0.73（95%CI: 0.57-0.88）。Mu等^[29]^纳入194例IIIB-IV期接受免疫治疗的NSCLC患者，从采集于免疫治疗开始6个月以内的氟代脱氧葡萄糖正电子发射计算机断层显像（^18^F-flurodeoxyglucose positron emission tomography/CT, ^18^F-FDG PET/CT）中提取影像组学特征建立列线图预测模型，结果显示在前瞻性验证集（n=48）中，列线图预测PFS和OS的C指数分别为0.77（95%CI: 0.69-0.84）和0.80（95%CI: 0.69-0.91）。以上诸多基于免疫治疗前平扫CT或增强CT、免疫治疗短期随访CT亦或^18^F-FDG PET/CT等建立影像组学预测模型的研究均证明了影像组学可以指导NSCLC患者更加个体化的诊疗决策。

此外，Yolchuyeva等^[40]^采用多种机器学习和特征筛选方法的不同组合进行多中心预后预测建模，论证了恰当的特征选择方法与机器学习策略相结合用于开发生存模型的重要性。Yoon等^[41]^探索影像组学特征与肿瘤微环境关系的研究同样证明了影像组学作为无创生物标志物的潜力。Peng等^[42]^采用11个亚区域的影像组学特征开发了分区域影像组学模型（sub-regional radiomics model, SRRM），结果显示SRRM相比常规影像组学、PD-L1表达和TMB评分具有更好的预测性能，并能对接受ICIs治疗的NSCLC患者PFS进行有效分层。

上述诸多国内外研究皆已证明影像组学对预测NSCLC患者免疫治疗反应具有重要作用，但受限于特征标准化、病症分割的高效性和可重复性及数据交叉验证等方面仍存在不足^[43]^，影像组学生物标志物应用于临床实践仍面临诸多困难与挑战。

### 2.3 辅助识别PsPD和超进展（hyperprogression, HPD）

免疫治疗反应表现复杂^[44]^，包括肿瘤病灶缩小、PsPD、HPD亚型和获得性耐药等。取决于肿瘤类型及评估标准，NSCLC免疫治疗后PsPD的发生率为0.6%-9.96%，HPD的发生率为5.0%-37.0%^[45⇓⇓-48]^。PsPD是免疫治疗过程中肿瘤组织的非典型反应，指满足RECIST的疾病进展，但活检证实为坏死或炎症细胞浸润，随后肿瘤负荷减轻^[49]^。Tazdait等^[50]^回顾性分析160例接受ICIs治疗的晚期NSCLC患者，在20例（13%）患者中观察到非典型反应，包括8例（5%）PsPD和12例（8%）分离反应，结果指出PsPD和分离反应患者总生存率高于真进展的患者，RECIST低估了发生PsPD中11%患者的ICIs获益情况。Eisenhauer等^[49]^指出RECIST将肿瘤大小变化作为唯一考量因素不能识别PsPD，存在低估ICIs治疗获益的缺陷。2017年RECIST指导小组制定实体瘤免疫治疗疗效评价标准（immune RECIST, iRECIST），iRECIST允许在首次发生PsPD时继续治疗并在4-8周内随访评估免疫治疗反应。Ahmed等^[51]^前瞻性纳入42例接受PD-1或PD-L1单抗治疗的晚期NSCLC患者，基于治疗前后PET/CT评估肿瘤负荷状态和患者肿瘤PFS及OS并比较RECIST、iRECIST及实体瘤PET反应标准（PET RCIST, PERCIST），研究指出三项疗效评价标准对免疫应答者和无应答者分层能力无显著差异。Chiou等^[52]^认为与常规化疗、靶向治疗相比，免疫治疗的肿瘤反应模式可能不同，通过影像资料难以准确把握免疫治疗反应。

Li等^[53]^回顾性分析来自三家机构105例接受免疫治疗的NSCLC患者，利用基线CT扫描所开发的影像组学预测模型在区分PsPD和HPD方面表现出最佳性能，在训练集和验证集的AUC分别为0.95（95%CI: 0.93-1.0）和0.75（95%CI: 0.72-1.0）；Kaplan-Meier生存曲线显示，模型预测的PsPD与真进展（肿瘤进展和HPD）在OS率上存在明显分层。研究证实了影像组学可以有效预测免疫治疗的NSCLC患者发生PsPD和HPD。Tunali等^[54]^纳入228例接受免疫单药或双药联合的NSCLC患者，利用合成少数群体过采样技术（synthetic minority oversampling technique, SMOTE）消除分类偏倚并构建临床影像组学复合模型，探索能够识别接受ICIs治疗但具有肿瘤快速进展高危风险患者的预测因子，结果显示模型对于肿瘤进展时间小于2个月或肿瘤快速进展的患者，其分类准确率分别为73.4%和82.3%，表现出良好的预测能力。Vaidya等^[28]^纳入109例接受免疫单药治疗的晚期NSCLC患者，使用随机森林法建立了能够识别具有HPD风险患者的影像组学模型。可以认为，影像组学的应用有助于识别免疫治疗后PsPD或具有HPD风险的患者，并指导此类患者精准治疗策略的制定。

准确识别PsPD非常关键，原发灶或转移灶的重复病理活检有助于诊断，已有诸多小样本研究^[55⇓-57]^证明血清学指标如中性粒细胞/淋巴细胞比值（neutrophil to lymphocyte ratio, NLR）、循环肿瘤DNA（circulating tumor DNA, ctDNA）、白介素8（interleukin 8, IL-8）和CXC趋化因子配体2（C-X-C motif chemokine ligand 2, CXCL2）等在鉴别PsPD方面具有良好的诊断潜力，但均因昂贵或有创性致临床应用受限。目前准确识别PsPD仍面临诸多困难，有关影像组学鉴别PsPD和HPD的研究甚少，未来仍需更多可靠的研究来证明影像组学在鉴别肿瘤PsPD和真进展中的潜力。

### 2.4 预测CIP

CIP^[58]^是一种潜在的致命性副作用，在接受ICIs治疗的NSCLC患者中发生率为4%-10%，需要准确诊断及大剂量糖皮质激素治疗。而接受免疫治疗的NSCLC患者同样可以发生许多其他类型肺炎（other types of pneumonitis, OTP），例如细菌、病毒或真菌感染，放射性肺炎（radiation-induced pneumonitis, RP）。根据最新发布的指南^[59]^，CIP的鉴别诊断尚无金标准。Tohidinezhad等^[60]^回顾性纳入荷兰和比利时多家机构共556例接受免疫治疗的晚期NSCLC合并肺炎的患者，其中31例（5.6%）发生CIP，41例（7.4%）发生OTP，以肺炎表现时扫描的CT图像建立影像组学模型，结果显示模型的ROC为0.83（95%CI: 0.77-0.95），阴性预测值和阳性预测值分别为80%和79%。Qiu等^[61]^回顾性纳入126例NSCLC合并肺炎患者，应用Logistic回归建立CT影像组学列线图预测模型以区分CIP和RP，模型在训练集和验证集中均表现出良好的鉴别能力。Wang等^[62]^回顾性纳入130例NSCLC患者，这些患者在接受单纯免疫治疗（ICIs组：n=50）、单纯放疗（radiotherapy, RT）（n=50）及免疫联合放疗（ICIs+RT组：n=30）后发生肺炎，研究建立CT影像组学模型用于鉴别肺炎病因，结果显示：与ICIs或RT组患者相比，ICIs+RT患者发生高级别（3-4级）肺炎更多，且联合治疗后发生的肺炎不是RP和CIP的简单叠加，研究同时指出最有助于鉴别RP和CIP的4个影像组学特征。然而，考虑到免疫治疗严重不良反应的相对稀少性，仍然需要更大规模的前瞻性研究进行综合评估。

## 3 预测恶病质风险

多项研究^[63⇓-65]^表明肿瘤恶病质对晚期NSCLC患者免疫化疗存在不利影响，约50%的癌症患者最终发展为恶病质，可能导致ICIs原发耐药的发生，Mu等^[66]^利用210例NSCLC患者免疫治疗前PET/CT图像提取影像组学特征预测恶病质风险，结果显示在训练集、测试集和外部测试集中影像组学特征预测ICIs恶病质风险的AUC≥0.74，研究证实了基于治疗前PET/CT影像组学特征可以作为潜在的预测性生物标志物用于识别接受ICIs治疗后可能发生恶病质的患者。目前影像组学模型预测NSCLC患者恶病质风险的相关研究较少，未来需要进一步的基础研究探讨恶病质对NSCLC患者免疫治疗的影响及具体机制。

## 4 关于多组学预测模型的探索

Bouhamama等^[67]^为预测晚期NSCLC免疫治疗反应，纳入54例患者（39例疾病控制，15例疾病进展）建立转录组学联合影像组学模型，其中75%的数据用于训练，25%用于验证，分别使用t-test、Wilcoxon、AUROC纳入1个影像组学特征和19个相关基因，使用ReliefF纳入4个影像组学特征和16个相关基因建立多组学模型，研究显示影像组学与基因组数据相结合可能提高模型的预测性能，但因为受试者数量少，不能肯定地说明多组学模型优于影像组学模型。CD274是PD-L1的编码基因，Chen等^[68]^基于CD274开发的基因组学联合影像组学的基因-影像组学模型（lung cancer immunotherapyradiomics prediction vector, LCI-RPV），共纳入194例NSCLC患者，其中85例可获得增强CT及CD274的患者作为训练集，两独立验证集分别包含66例和43例接受ICIs治疗的NSCLC患者，结果显示LCI-RPV能够预测免疫治疗后3个月的肿瘤免疫反应（AUC=0.68, 95%CI: 0.52-0.85）及肺炎发生率（AUC=0.64, 95%CI: 0.48-0.80），并将患者分为高风险和低风险生存组（风险比=2.26，95%CI: 1.21-4.24，P=0.011；风险比=2.45，95%CI: 1.07-5.65，P=0.035）。免疫相关不良反应在免疫治疗应答者中富集^[69]^，这提示着我们LCI-RPV可能用于识别部分ICIs治疗前预先存在T细胞自动活化的患者。未来的多组学预测模型仍需在更长的随访周期和更大数据集中证明其在患者免疫治疗反应及预后中的预测价值。

## 5 总结与展望

影像组学能够无创、动态地提取整个肿瘤及瘤周区域诊断和治疗全过程的生物学信息，亟待常规应用于癌症分期、治疗反应评估、随访和不良反应管理全过程。不断探索新型预后生物标志物以指导临床实践也是精准医学的内在要求，目前越来越多的研究聚焦于多组学联合预测模型的探索。然而，目前的影像组学研究质量有待提高，研究结果存在异质性且缺乏深入的模型验证。将影像组学模型应用于临床实践中仍需要开展更多纳入外部验证集的前瞻性研究以开发出预测性能更优、推广性更好的复合模型。总之，更多前瞻性、多中心、大规模的随机对照临床试验仍需国内外学者们的共同努力。
